# Investigating the Role of Viruses in the Rapid Decline of Young Apple Trees in High-Density Orchards in New York

**DOI:** 10.3390/plants13202866

**Published:** 2024-10-14

**Authors:** Anna O. Wunsch, Mario Miranda Sazo, Janet van Zoeren, Kurt H. Lamour, Oscar P. Hurtado-Gonzales, Awais Khan, Marc Fuchs

**Affiliations:** 1 Plant Pathology and Plant-Microbe Biology Section, School of Integrative Plant Science, Cornell University, Geneva, NY 14456, USA; awais.khan@cornell.edu (A.K.); marc.fuchs@cornell.edu (M.F.); 2 Cornell Cooperative Extension Lake Ontario Fruit Program, Albion, NY 14411, USA; mrm67@cornell.edu (M.M.S.); jev67@cornell.edu (J.v.Z.); 3 Department of Entomology and Plant Pathology, University of Tennessee, Knoxville, TN 37996, USA; klamour@utk.edu; 4 USDA-APHIS Plant Germplasm Quarantine Program, Beltsville, MD 20705, USA; oscar.hurtado-gonzales@usda.gov

**Keywords:** latent apple virus, apple stem pitting virus, apple chlorotic leaf spot virus, apple stem grooving virus, high-density orchard, rapid apple decline

## Abstract

A sudden, unexplained decline and collapse of young apple trees on dwarfing and semi-dwarfing rootstocks has been reported across North America over the past decade. Although viruses have been detected in declining trees, no information is available on their potential causal role in the decline phenomenon. To this end, virus-inoculated apple trees were established in a high-density experimental orchard and monitored over five years. Tree decline was observed in year 4 (2022), resulting in 17% mortality, with declining trees exhibiting marked vascular tissue necrosis. However, none of the eight viruses and one viroid detected in the experimental orchard was significantly more prevalent in declining trees. Extreme temperature fluctuations in January 2022, followed by a severe water deficit in summer 2022, were recorded at the experimental orchard. Similar but distinct observations were made in a nearby commercial orchard with foliar nutrient imbalances documented in trees exhibiting symptoms of rapid decline. Together, our findings suggest that viruses are not primarily responsible for the rapid decline phenomenon and highlight the need for future work to investigate the roles of tree physiology and water stress in tree decline, as well as the potential efficacy of horticultural mitigation practices.

## 1. Introduction

A sudden, unexplained, mid-season collapse of young apple trees (*Malus domestica* Borkh.) in high-density orchards, which has been referred to as “rapid apple decline” or “sudden apple decline”, was first reported in the eastern United States in the 2010s [1,2,3,4]. Similar sudden, unexplained apple tree decline phenomena have since been reported in New York [3,5,6], North Carolina [4], Pennsylvania [1], Washington [7], and West Virginia [8] in the United States, as well as in British Columbia [9] in Canada.

These decline phenomena are characterized by the rapid collapse of young apple trees in high-density plantings over the course of several weeks, often resulting in tree mortality [1,2,8]. Tree collapse is sometimes preceded by a gradual decline of tree health over the course of a growing season, typified by chlorosis throughout the canopy and declining tree vigor [1,2,10,11]. Trees which collapse typically do so during the active growing season, often with a full crop load [1,2,4,6,11]. The underlying causal factors of the decline phenomena are yet to be identified, although many have been hypothesized, including fire blight of the rootstock caused by the bacterial pathogen *Erwinia amylovora*, herbicide injury, boring insects, winter injury, drought stress, fungal canker and wood rot fungi, and latent viruses [3,5,6,8,9,11,12]. This rapid mid-season collapse of young apple trees has not been attributed to a known disease, such as apple bacterial quick decline in Japan caused by *Dickeya dadantii* [13] or stem canker and dieback in Ontario caused by *Botryosphaeria dothidea* [14].

Several recent studies have attempted to assess the role of viruses and viroids in the decline phenomenon. In Pennsylvania, high-throughput sequencing revealed the presence of a novel luteovirus, i.e., apple luteovirus 1 (ALV1), in declining trees, as well as three common latent viruses of apple, i.e., apple chlorotic leaf spot virus (ACLSV), apple stem pitting virus (ASPV), and apple stem grooving virus (ASGV) [8]. Latent apple viruses generally do not elicit symptoms on commercial cultivars and rootstocks, often occur in mixed infections, and are endemic to cultivated apple trees [15]. A comprehensive study in a commercial orchard in New York detected two latent viruses, i.e., ACLSV and ASPV, in both declining and non-declining trees [6]. In Washington, high-throughput sequencing revealed the presence of several novel putative viruses, as well as nine known viruses and viroids, in both declining and non-declining trees in commercial orchards, but none was consistently associated with decline [12]. In British Columbia, high-throughput sequencing and molecular assays identified a novel ilarvirus, i.e., apple ilarvirus 2, as well as 21 known viruses and viroids, in both declining and non-declining trees in commercial orchards [9].

In addition to investigating the role of viruses and viroids in decline phenomena, studies conducted to date have also investigated plant and soil microbial community composition [6], weather patterns [6,16], tree water status [11], and root system architecture [10]. Singh et al. (2019) used high-throughput sequencing to identify the composition of bacterial and fungal communities in apple root, shoot, rhizosphere, and soil samples and documented no significant enrichment of any class of bacteria or fungi in declining trees in a commercial apple orchard in western New York [6]. However, this study identified unusually cold winter temperatures followed by summer drought in the vicinity of the declining orchard [6]. Similarly, Donahue & Elone (2021) documented uncharacteristically variable temperatures in their case study of a declining commercial orchard in eastern New York [16]. A comparison of the root system architectures of declining and non-declining trees in two commercial orchards in New York found that the root systems and scion trunk diameters of declining trees were significantly smaller than non-declining trees in one orchard but not the other [10]. Furthermore, a study of the water status of trees in a declining orchard in British Columbia found that tree decline and mortality were associated with disruption in xylem water transport and hydraulic failure [11].

All studies conducted to date which have investigated the role of viruses and viroids in the rapid decline of young apple trees have represented observational surveys of trees already in decline at a single point in time during the life of an orchard [6,8,9,10,12]. To our knowledge, no study to date has assessed the potential role of viruses and viroids in the decline phenomenon by monitoring declining trees for more than one growing season. The objectives of this study were to monitor the progress of decline in an experimental orchard plot beginning at orchard establishment, to reproduce the rapid decline phenomenon under experimental conditions, and to conclusively rule latent virus infections in or out as a primary cause while simultaneously monitoring the progress of decline in a nearby commercial orchard block. We hypothesized that concurrent infection by a greater number of viruses would result in higher rates of tree decline at both sites. Here, we summarize our efforts to characterize the rapid decline phenomenon in both a high-density experimental apple orchard and a high-density commercial apple orchard in the Lake Ontario fruit production region of New York.

## 2. Results

### 2.1. Observations of Rapid Tree Decline in an Experimental Apple Orchard

An experimental apple orchard was established in 2019 at Cornell AgriTech in Ontario County, NY, USA, in an effort to reproduce the rapid decline phenomenon. Trees representing six scion-rootstock combinations (‘Baigent’ Gala, ‘Honeycrisp’, and ‘Royal Red Honeycrisp’™ (Willow Drive Nursery, WA, USA) on ‘Malling 26’ (M.26) or ‘Geneva 935’ (G.935) rootstocks) were custom-budded the year prior and then chip graft inoculated with either apple chlorotic leaf spot virus (ACLSV), apple stem pitting virus (ASPV), both viruses, or neither virus. Over the course of the five-year study, 65 of the 240 total trees established in the experimental orchard were lost for reasons unrelated to the tree decline phenomenon of interest, including unsuccessful budding, tree establishment failure, and fire blight caused by *Erwinia amylovora* infection. These trees were excluded from the study.

**Figure 1:**
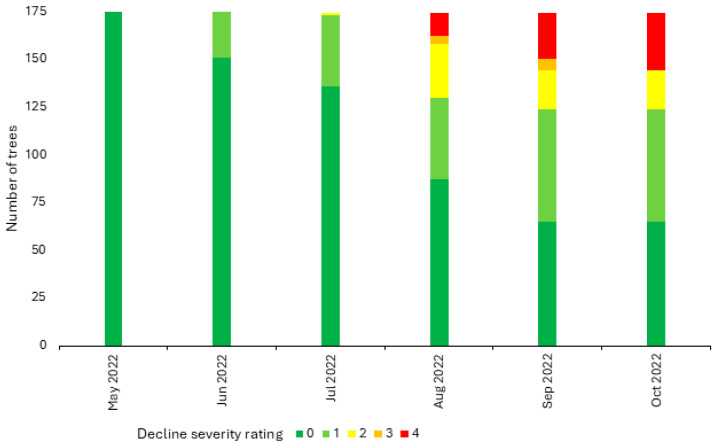
Decline severity ratings over time of three-year-old ‘Baigent’ Gala, ‘Honeycrisp’, and ‘Royal Red Honeycrisp’™ apple trees on ‘Malling 26’ and ‘Geneva 935’ rootstocks in a high-density experimental orchard at Cornell AgriTech in Ontario County, NY, USA over the course of the 2022 growing season. Tree decline severity was visually assessed monthly from May to October using a scale from 0 to 4, with a rating of 0 indicating no decline symptoms and a rating of 4 indicating tree mortality. Ratings of 1 to 3 indicate increasing decline severity, with a rating of 1 corresponding to chlorosis throughout <50% of the canopy, a rating of 2 corresponding to chlorosis throughout ≥50% of the canopy, and a rating of 3 corresponding to chlorosis throughout ≥50% of the canopy, leaf flagging, and poor tree vigor.

**Figure 2:**
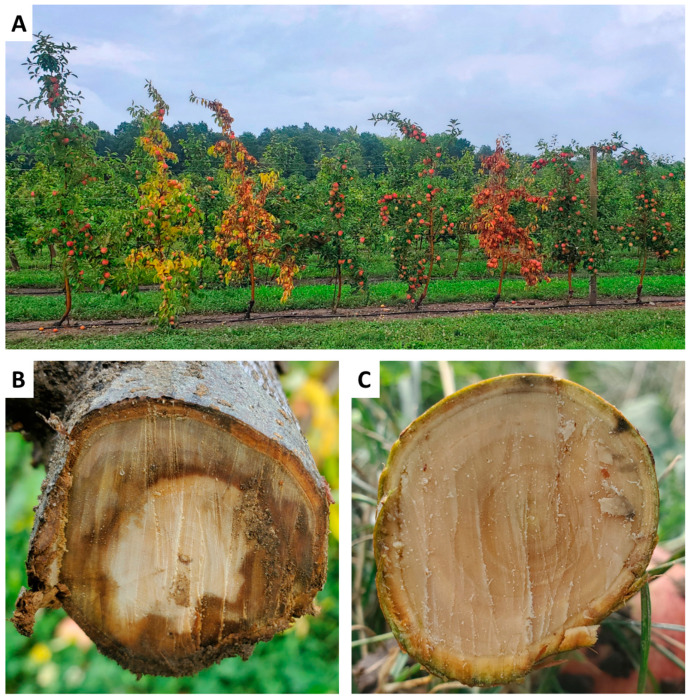
(**A**) Asymptomatic, declining, and collapsed three-year-old ‘Baigent’ Gala, ‘Honeycrisp’, and ‘Royal Red Honeycrisp’™ apple trees on ‘Malling 26’ and ‘Geneva 935’ rootstocks in a high-density experimental orchard at Cornell AgriTech in Ontario County, NY, USA on 22 August 2022. Tree decline severity was visually assessed using a scale from 0 to 4, with a rating of 0 indicating no decline symptoms and a rating of 4 indicating tree mortality. From left to right, the eight trees pictured here represent decline severity ratings of 0, 3, 4, 0, 0, 4, 0, and 0, respectively; (**B**) A representative cross-section of the trunk of a severely declining tree (decline severity rating 3) showing extensive browning and necrosis of the vascular tissue approximately 30 cm above the graft union. Cross-sections of 15 declining trees were evaluated (four with a decline severity rating of 3; 11 with a decline severity rating of 4), and all displayed similar tissue necrosis; (**C**) a representative cross-section of the trunk of a non-declining tree (decline severity rating 0) without browning or necrosis of the vascular tissue approximately 30 cm above the graft union. Cross-sections of two non-declining trees were evaluated (both had decline severity ratings of 0), and both displayed a similar lack of tissue necrosis.

The decline severity of the remaining 175 trees (175 of 240; 73%) was visually evaluated according to a 0 to 4 severity scale from 2019 to 2023 (years 1–5). No tree declined (decline severity rating 0) in 2019, 2020, or 2021 (years 1–3). In 2022 (year 4), all 175 trees successfully emerged from dormancy, bloomed, and set fruit (decline severity rating 0) (Figure 1). Interestingly, some trees began showing initial signs of decline, corresponding to decline severity rating 1, in early June (approximately four weeks post-bloom) (Figure 1). By early August (approximately 12 weeks post-bloom), trees with symptoms corresponding to all five decline severity ratings were observed, including complete tree collapse (decline severity rating 4) in three trees (1.7%, 3 of 175) (Figure 1 and Figure 2). By the onset of leaf senescence in mid-October (approximately 22 weeks post-bloom), the tree mortality rate (decline severity rating 4) reached 17% (30 of 175) (Figure 1). All three cultivars and both rootstocks evaluated were represented in the population of 30 trees which collapsed in 2022: ‘Baigent’/G.935 (*n* = 2), ‘Baigent’’/M.26 (*n* = 15), ‘Honeycrisp’/G.935 (*n* = 1), ‘Honeycrisp’/M.26 (*n* = 11), and ‘Royal Red Honeycrisp’™/G.935 (*n* = 1). No leaf drop or substantial trunk swelling or cracking at the graft union was apparent in any tree. However, cross-sectioning of the trunks of severely declining trees (decline severity rating 3) revealed extensive browning and necrosis of the vascular tissue (Figure 2). Together, this work documented the occurrence of the rapid decline phenomenon in the experimental orchard across all evaluated apple cultivars and rootstocks in year 4. The rapid decline was accompanied by vascular tissue necrosis.

Overall, the majority of the trees evaluated (62%, 109 of 175) experienced either mild (decline severity rating 1), moderate (decline severity rating 2) or severe decline (decline severity rating 3) during the 2022 growing season (Figure 1). The 30 trees that collapsed (decline severity rating 4) made up 28% (30 of 109) of those which showed symptoms of decline (Figure 1). Strikingly, none of the surviving 145 trees declined in 2023 (year 5), including those showing signs of mild or moderate decline at the end of the 2022 growing season (45%, 79 of 175).

### 2.2. Detection of Viruses and Viroids in the Experimental Apple Orchard

Diagnostic testing of 175 trees in the experimental orchard via multiplex polymerase chain reaction (PCR)-based amplicon sequencing revealed the presence of eight viruses and one viroid (Table 1). At least one virus or viroid was identified in every tree tested (Table 1 and Table S1). The three most prevalent viruses were apple stem grooving virus (ASGV) (100%, 175 of 175), ACLSV (78%, 136 of 175), and ASPV (71%, 125 of 175) (Table 1). Five additional viruses, i.e., citrus concave gum-associated virus (CCGaV), apple green crinkle-associated virus (AGCaV), apple rubbery wood virus 2 (ARWV2), tobacco ringspot virus (TRSV), and tomato ringspot virus (ToRSV), as well as one viroid, apple hammerhead viroid (AHVd), were detected in smaller proportions of the trees tested (Table 1 and Table S1). Ten of the viruses and viroids detectable by this methodology were not identified in the experimental orchard (Table 1 and Table S1). This work indicated the presence of more viruses and viroids than expected based on the initial design of the experimental orchard that relied on chip graft inoculation of selected trees with either ACLSV, ASPV, or both viruses.

### 2.3. The Relationship between Virus Infection and Decline Outcome in the Experimental Apple Orchard

**Figure 3:**
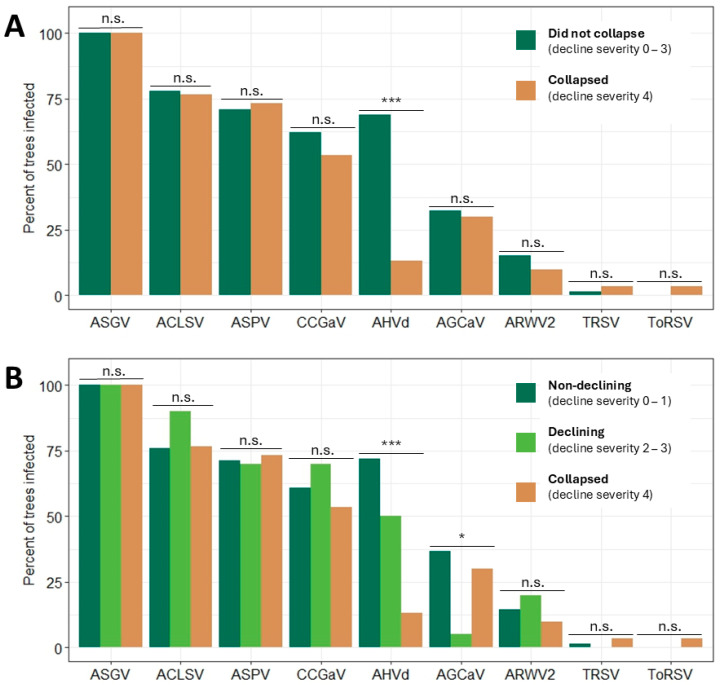
Prevalence of eight viruses and one viroid in 175 three-year-old ‘Baigent’ Gala, ‘Honeycrisp’, and ‘Royal Red Honeycrisp’™ apple trees on ‘Malling 26’ and ‘Geneva 935’ rootstocks in a high-density experimental orchard at Cornell AgriTech in Ontario County, NY, USA that (**A**) did or did not collapse in 2022, or (**B**) collapsed, declined, or did not decline in 2022. Decline severity ratings were assigned as previously described. Statistical significance was determined by χ^2^ tests with a *p* value threshold of 0.05. n.s. *p* ≥ 0.05, * *p* < 0.05; *** *p* < 0.001. Abbreviations: ACLSV, apple chlorotic leaf spot virus; AGCaV, apple green crinkle-associated virus; AHVd, apple hammerhead viroid; ARWV2, apple rubbery wood virus 2; ASGV, apple stem grooving virus; ASPV, apple stem pitting virus; CCGaV, citrus concave gum-associated virus; ToRSV, tomato ringspot virus; TRSV, tobacco ringspot virus.

The relationship between virus and viroid prevalence in collapsed trees (decline severity rating 4; *n* = 30) and in trees that did not collapse (decline severity ratings 0, 1, 2, or 3; *n* = 145) was evaluated for statistical significance (Figure 3A). Of the eight viruses and one viroid detected in the experimental orchard, none was found to be significantly more prevalent in trees that collapsed compared with trees that did not collapse (Figure 3A). Furthermore, AHVd prevalence was significantly higher in trees that did not collapse (69%, 100 of 145) than in trees that did collapse (13%, 4 of 30) (*p* < 0.001) (Figure 3A). Moreover, an analysis of variance revealed no statistically significant difference between the number of unique viruses and viroids detected in trees that collapsed ($\overset{-}{x}$ = 3.63) and in trees that did not collapse ($\overset{-}{x}$ = 4.29) (*p* = 0.062), revealing no association between tree mortality and the number of unique viruses and viroids detected.

Similarly, of the eight viruses and one viroid detected in the experimental orchard, none was found to be significantly more prevalent in declining trees (decline severity ratings 2–3; *n* = 20) or trees that had collapsed (decline severity rating 4; *n* = 30) compared with non-declining trees (decline severity ratings 0–1; *n* = 125) (Figure 3B). Indeed, the prevalences of both AHVd and AGCaV were significantly higher in non-declining trees (72% and 37%, respectively) than in declining trees (50% and 5%; *p* < 0.001 and *p* = 0.034, respectively) (Figure 3B). Together, these results revealed that the prevalence of common apple viruses and viroids was not associated with tree decline outcome in the experimental orchard.

Because the viruses and viroid detected in the experimental orchard were present in many unique combinations, the number of trees infected with any one combination was generally quite small, precluding a robust statistical analysis of correlation between virus combinations and decline outcome. Nevertheless, we observed no clear association between particular virus combinations and tree decline or collapse (Table S1).

### 2.4. Root System Architecture of Trees in the Experimental Apple Orchard

Root system architecture parameters were compared between excavated root systems of trees that experienced substantial decline (decline severity ratings of 2, 3, or 4; *n* = 25) and those that did not experience substantial decline (decline severity ratings of 0 or 1; *n* = 14). Of the root system parameters evaluated, both scion diameter and rootstock diameter at the graft union differed significantly between scion cultivars (*p* = 0.001 and *p* = 0.025, respectively) (Table S2). These differences are attributable to known differences in trunk cross-sectional area and overall tree size between ‘Gala’ and ‘Honeycrisp’ cultivars, as previously described [17]. No other significant differences in the root system architecture parameters evaluated were observed based on cultivar or rootstock (Table 2, Tables S2 and S3). Although none of the root system parameters evaluated differed significantly between declining and non-declining trees when a *p* value threshold of 0.05 was applied, root system depth was significantly shallower in trees that experienced substantial decline than in trees that did not experience substantial decline when a *p* value threshold of 0.10 was applied instead (*p* = 0.058), possibly due to the high root system depth variability observed (Table 2). Although these data must be interpreted with caution, they may indicate reduced root depth in declining trees.

### 2.5. Weather Patterns at the Experimental Apple Orchard Site

**Figure 4:**
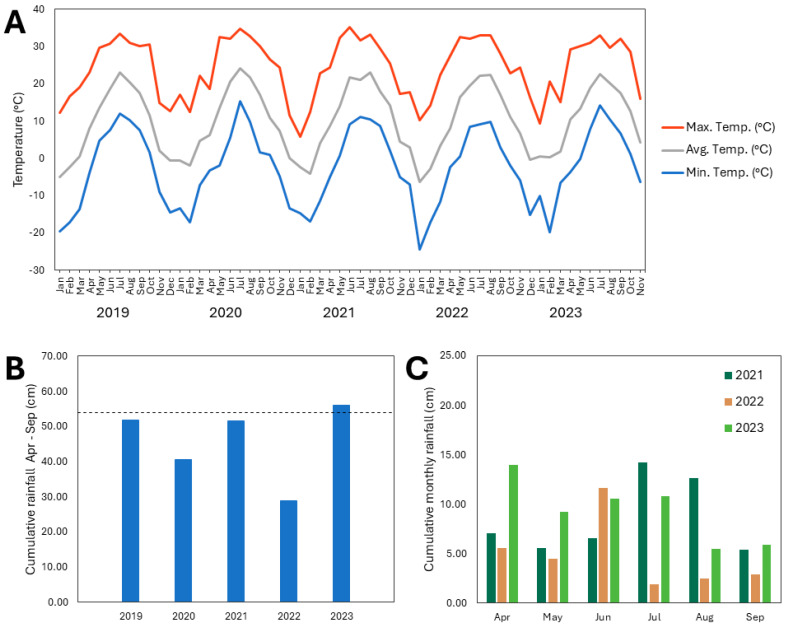
(**A**) Maximum, average, and minimum temperatures each month from January 2019 to November 2023; (**B**) cumulative rainfall during each growing season (April–September) from 2019 to 2023, where the dashed line indicates the twenty-year average rainfall (2003–2023) received during April–September in this region; (**C**) cumulative rainfall each month of the growing season (April–September) from 2021 to 2023 at the site of the experimental apple orchard at Cornell AgriTech in Ontario County, NY, USA.

Weather data analysis indicated that between 2019 and 2023, the rainfall received at the experimental orchard site was below average for the region every year except for 2023, based on a 20-year rainfall average of 54.0 cm for these months (Figure 4) [18]. The site received significantly less rainfall in 2022 than in other years, with cumulative rainfall from April to September of only 29.0 cm (Figure 4). The rainfall deficit was particularly pronounced during July, August, and September 2022 (Figure 4). During these three months, the orchard site received only 7.4 cm of rainfall (compared with 24.1, 24.1, 32.3, and 22.4 cm during the same months in 2019, 2020, 2021, and 2023, respectively) (Figure 4).

Additionally, the orchard site experienced more extreme temperature fluctuations in January 2022 compared with the other years of the study, with the lowest minimum temperature of −24.5 °C (compared to the minimum January temperatures of −19.7, −13.4, −14.7, and −10.2 °C in 2019, 2020, 2021, and 2023, respectively), as well as the greatest temperature range, with a total difference between maximum and minimum temperatures of 34.8 °C in January 2022 (compared to January temperature ranges of 31.9, 30.5, 20.4, and 19.6 °C in 2019, 2020, 2021, and 2023, respectively) (Figure 4).

### 2.6. Water Balance of Trees at the Experimental Apple Orchard Site

**Figure 5:**
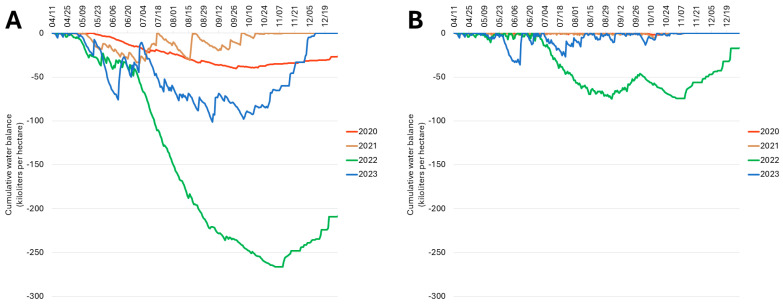
Cumulative orchard water balance in a high-density experimental apple orchard at Cornell AgriTech in Ontario County, NY, USA, from 2020 to 2023 (**A**) without accounting for supplemental irrigation; and (**B**) accounting for supplemental irrigation. The cumulative orchard water balance is expressed in kiloliters per hectare.

Modeling the water balance of trees in the experimental orchard indicated that the water demand of rainfed trees was higher than the rainfall received each growing season, with maximum negative water balances of −40,451, −33,999, −266,206, and −101,015 L per hectare in 2020, 2021, 2022, and 2023, respectively (Figure 5A). However, the drip irrigation provided was sufficient to satisfy orchard water demand every year of the study, with the exception of 2022 (Figure 5B). When accounting for irrigation, the experimental orchard had an estimated maximum negative water balance of −74,676 L per hectare on 4 September 2022. These analyses revealed a substantial water deficit of trees at the experimental apple orchard site when decline was documented in the summer of 2022.

### 2.7. Observations of Rapid Tree Decline in a Commercial Apple Orchard

In addition to monitoring decline severity in the experimental orchard, approximately 600 ‘Honeycrisp’/‘Malling 9 Nic29’ trees planted in a commercial orchard in Wayne County, NY, USA, in 2018 were visually evaluated according to the same 0 to 4 decline severity scale during the 2021–2023 growing seasons (years 4–6). The commercial orchard site was located 35 km from the experimental orchard and was selected for this study after the orchardist noticed suddenly declining trees in 2020 (year 3). 

**Figure 6:**
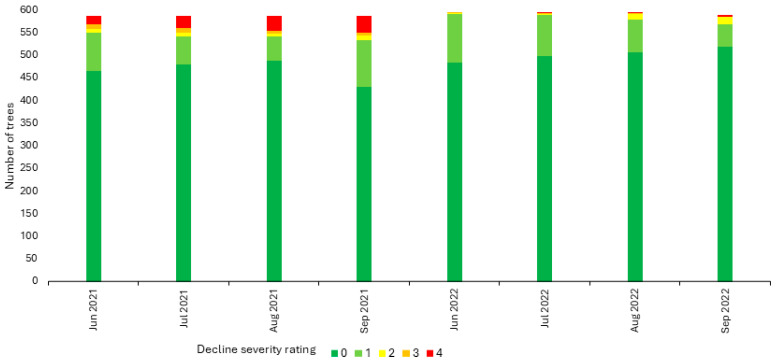
Decline severity over time of ‘Honeycrisp’ apple trees on ‘Malling 9 Nic29’ rootstocks established in 2018 in a high-density commercial orchard block in Wayne County, NY, USA, over the course of the 2021 and 2022 growing seasons (June–September). Tree decline severity was visually assessed using a scale from 0 to 4, with a rating of 0 indicating no decline symptoms and a rating of 4 indicating tree mortality. Ratings of 1 to 3 indicate increasing decline severity, with 1 corresponding to chlorosis throughout <50% of the canopy, 2 to chlorosis throughout ≥50% of the canopy, and 3 to chlorosis throughout ≥50% of the canopy, leaf flagging, and poor tree vigor.

In 2021 (year 4), all trees in the commercial orchard block successfully emerged from dormancy, bloomed, and set fruit (decline severity rating 0). At the first decline severity assessment in June (approximately seven weeks post-bloom), trees with symptoms corresponding to all five decline severity ratings were observed, including complete tree collapse (decline severity rating 4) in 20 trees (3.4%, 20 of 588) (Figure 6). By late September (approximately 20 weeks post-bloom), the tree mortality rate (decline severity rating 4) had reached 6.5% (38 of 588) (Figure 6). Trees that collapsed during the 2021 season were removed and replaced with new trees by the orchardist that autumn.

The impact of rapid decline in the commercial orchard was significantly less severe in 2022 (year 5). All surviving trees successfully emerged from dormancy, bloomed, and set fruit (decline severity rating 0), including those which had exhibited mild to moderate decline severity the previous year. At the first decline severity assessment in June (approximately four weeks post-bloom), some trees exhibited decline symptoms (decline severity ratings 1, 2, and 3), but none had collapsed (decline severity rating 4) (Figure 6). By September (approximately 21 weeks post-bloom), the tree mortality rate reached only 0.7% (4 of 594), representing a ten-fold decrease in mortality compared to the previous year. Moreover, none of the replacement trees planted the previous year exhibited decline symptoms in 2022. The few trees that collapsed during the 2022 growing season were removed and replaced with new trees by the orchardist that autumn. In 2023 (year 6), no trees declined, including those replaced in 2021 and 2022. No leaf drop or significant trunk swelling or cracking at the graft union was apparent in any tree of the commercial orchard over the course of the study. Together, these results documented the occurrence of extensive rapid decline in the commercial orchard block in year 4, followed by a reduced decline incidence in year 5 and zero decline incidence in year 6.

### 2.8. Weather Patterns at the Commercial Apple Orchard Site and Water Balance of Trees 

**Figure 7:**
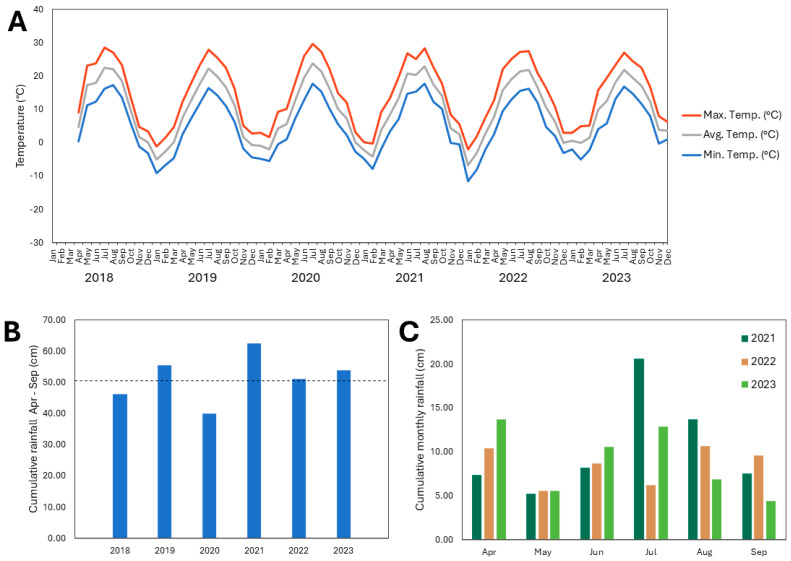
(**A**) Maximum, average, and minimum temperatures each month from March 2018 to December 2023; (**B**) cumulative rainfall during each growing season (April–September) 2018–2023, where the dashed line indicates the twenty-year average rainfall (2003–2023) received during April–September in this region; and (**C**) cumulative rainfall each month of the growing season (April–September) 2021–2023 near the site of a commercial apple orchard in Wayne County, NY, USA.

An analysis of weather patterns at the commercial orchard site was conducted using data obtained from a nearby weather station. The trees in this orchard block were not supplied with drip irrigation to supplement rainfall. Temperatures never rose above 30 °C in the summer or fell below −15 °C in the winter over the duration of the study (Figure 7), as is typical for this region due to the moderation of seasonal temperature extremes by the proximal Lake Ontario [18]. Monthly temperature ranges were also greatly moderated, with an average monthly temperature range of a mere 9.6 °C between monthly maximum and minimum temperatures at the commercial orchard site, as is typical for this region (Figure 7) [18].

Between 2018 and 2023, the commercial orchard site received the most rainfall (62.4 cm) during the 2021 growing season and the least (39.9 cm) during the 2020 growing season (Figure 7). The rainfall received during the growing season was below average for the region in 2018 and 2020 but was at or above average in 2019, 2021, 2022, and 2023, based on a 20-year rainfall average of 50.4 cm for these months (Figure 7) [18].

**Figure 8:**
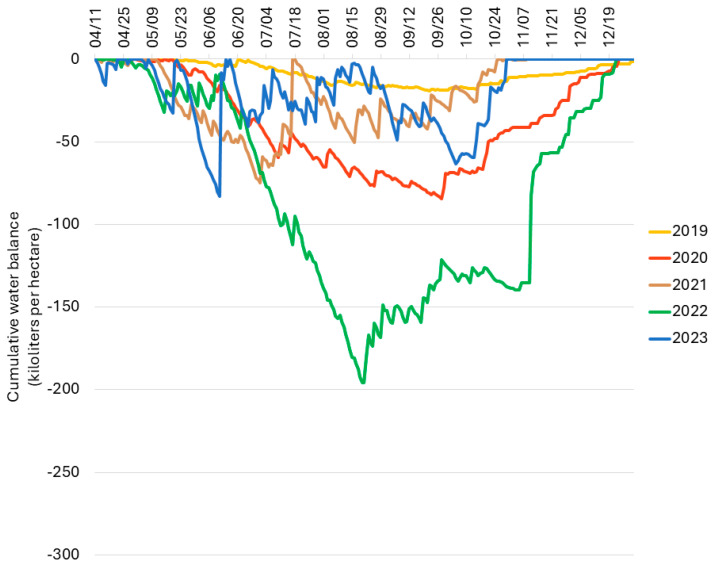
Cumulative orchard water balance in an unirrigated high-density commercial apple orchard in Wayne County, NY, USA, 2019–2023. The cumulative orchard water balance is expressed in kiloliters per hectare.

Modeling the water balance of trees at the commercial apple orchard site indicated that the water demand of rainfed trees was higher than the rainfall received each growing season, with maximum negative water balances of −19,342, −84,538, −75,039, −195,786, and −83,104 L per hectare in 2019, 2020, 2021, 2022, and 2023, respectively (Figure 8). Orchard water deficit was therefore greatest by far in 2022 and least in 2019 (Figure 8).

### 2.9. Foliar Nutrient Status of Trees in the Commercial Apple Orchard

**Figure 9:**
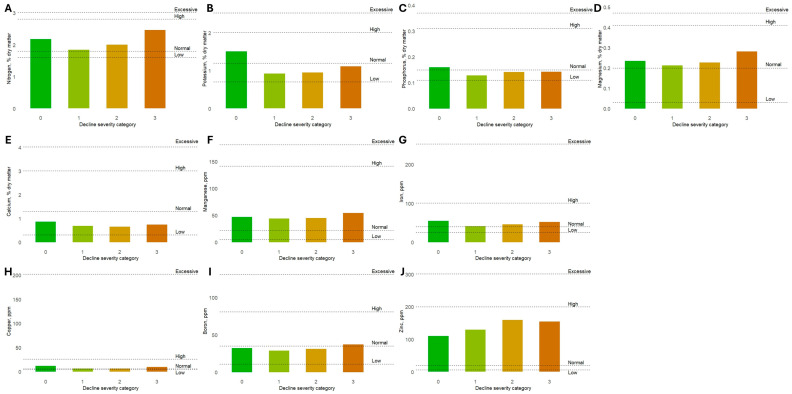
Foliar dry matter nutrient concentrations of (**A**) nitrogen, (**B**) potassium, (**C**) phosphorus, (**D**) magnesium, (**E**) calcium, (**F**) manganese, (**G**) iron, (**H**) copper, (**I**) boron, and (**J**) zinc in three-year-old ‘Honeycrisp’ apple trees on ‘Malling 9 Nic29’ rootstocks representing increasing decline severity. Thresholds indicating “Low”, “Normal”, “High”, and “Excessive” mineral nutrient concentrations in cultivated apple according to Pennsylvania State University nutrient guidelines are indicated by dashed lines [19].

Foliar nutrient analysis of trees in the commercial orchard in August 2021 (year 4) demonstrated that leaf elemental nitrogen, magnesium, manganese, iron, copper, and zinc concentrations were in the normal ranges for all four decline severity categories evaluated (decline severity ratings 0, 1, 2, and 3) (Figure 9A,D,F–H,J). However, leaf elemental calcium concentrations were in the low range for all four decline severity categories evaluated, possibly indicating an insufficient level of biologically available calcium at the orchard site (Figure 9E). Notably, the leaf elemental concentrations of potassium, phosphorous, and boron differed by decline severity category (Figure 9B,C,I). Both potassium and phosphorous were present in leaf tissue at concentrations in the normal ranges for non-declining trees (decline severity category 0) but at concentrations in the low ranges for mildly, moderately, and severely declining trees (decline severity ratings 1, 2, and 3) (Figure 9B,C). Conversely, boron was present in leaf tissue at concentrations in the low range for non-declining trees (decline severity category 0) and mildly and moderately declining trees (decline severity ratings 1 and 2) but at a concentration in the normal range for severely declining trees (decline severity rating 3) (Figure 9I). Together, these foliar nutrient analyses revealed nutrient imbalances in declining trees in comparison with non-declining trees.

## 3. Discussion

To our knowledge, this study represents the first investigation in which the rapid decline of young apple trees was recapitulated under experimental conditions. Prior attempts to characterize the decline phenomenon have solely comprised observational studies in commercial orchards. In this study, tree decline occurred in the Cornell AgriTech experimental orchard only in 2022, during the fourth growing season following orchard establishment, when full fruiting capacity was achieved for the first time. The observed decline symptoms were consistent with those previously reported in commercial apple orchards, with initial tree decline symptoms developing between June and September (between 4 and 18 weeks post-bloom), while the collapse of severely declining trees occurred between August and October (between 12 and 22 weeks post-bloom). Although nearly one-fifth (17%) of the evaluated trees collapsed, the decline symptoms of many mildly and moderately declining trees appeared to stabilize, never advancing to severe decline and collapse. All of the evaluated trees in decline severity categories 1 or 2 at the end of the 2022 growing season successfully emerged from dormancy the following spring, apparently having recovered.

A similar phenomenon, in which a considerable proportion of declining trees appeared to stabilize at mild or moderate decline stages rather than proceeding to severe decline and collapse, was observed in a declining commercial Zestar!™ (cv. ‘Minnewashta’, University of Minnesota, Minneapolis, MN, USA) apple orchard in the Hudson Valley production region of New York [16]. The distinction between gradual, reversible tree decline and abrupt tree collapse was explored by Xu et al. (2023) in a multi-site study of declining orchards in British Columbia [11]. In that study, assessments of stem hydraulic characteristics, stomatal conductance, carbon isotope content, and fruit dry matter accumulation revealed an association between tree mortality and severe xylem water transport disruption, while trees with symptoms of gradual decline exhibited moderate hydraulic dysfunction followed either by continued decline or eventual recovery [11]. Although stem hydraulic characteristics were not assessed in this study, our observations corroborate the patterns previously reported [11,16]. The leaf flagging and vascular necrosis observed in declining trees in the experimental orchard (Figure 2) appear to signal a vascular transport disruption. Similar trunk necrosis was previously reported in the commercial orchard monitored in this study [10], as well as in other orchards in New York [6] and in British Columbia [11].

The tree decline phenomenon observed in the Wayne County commercial orchard progressed similarly to that described above in the Cornell AgriTech experimental orchard. Decline symptoms observed were consistent with those previously reported in other commercial apple orchards. Initial symptoms of tree decline primarily developed between June and September each year; however, a subset of trees had already progressed to advanced stages of decline by the time of our first visit in June 2021 (year 4), suggesting that initial symptom development had begun earlier in the season. A similar distinction between abrupt tree collapse and gradual tree decline followed by either symptom stabilization and recovery or further decline, as discussed above, was observed in the commercial orchard block. No severely declining tree (decline severity rating 3) survived past the end of the growing season. However, many declining trees appeared to stabilize at a mild or moderate decline (decline severity ratings 1 and 2) and, after successful emergence from dormancy the following spring, did not display decline symptoms in the following growing seasons. Tree decline in the commercial orchard was most prevalent during the fourth growing season following orchard establishment (2021) and much less prevalent the following year (2022). No decline symptoms were observed in 2023, including in trees planted in 2021 and 2022 to replace those which had collapsed.

Multiplex PCR-based amplicon sequencing revealed the presence of eight viruses and one viroid in frequent mixed infections in the experimental orchard, with up to seven unique viruses and viroids detected in a single tree. These results indicated the presence of more viruses and viroids than expected based on the initial design of the experimental orchard that relied on chip graft inoculation of selected trees with either ACLSV, ASPV, or both viruses. We suspect that this was a result of undetected virus and viroid infection of the rootstock liners or scionwood sourced for this study. Virus testing by multiplex PCR-based amplicon sequencing also revealed the lack of uninfected trees in the experimental orchard, signifying a major limitation of our work due to the absence of uninfected trees to be used as negative controls. However, of the eight viruses and one viroid detected, all but ToRSV were detected in both declining and non-declining trees, as well as in trees which did not collapse and those which did. None of the eight viruses or viroid detected was more prevalent in trees which declined or in trees which collapsed entirely, leading us to reject our hypothesis that one or multiple viruses or viroids would be significantly more prevalent in declining trees. Surprisingly, the prevalence of AHVd was significantly higher in non-declining than in declining trees, as well as in trees which did not collapse versus those which did. As AHVd was detected primarily in trees on G.935 rootstocks, we suspect that this viroid was present in the G.935 rootstock liners sourced for this study. Similarly, three common apple latent viruses, i.e., ACLSV, ASPV, and ASGV were detected at similar rates in both declining and non-declining trees in the Wayne County commercial orchard, as previously reported [10]. Together, our results corroborate the findings of previous studies in Pennsylvania [8], New York [6], Washington [12], and British Columbia [9] by demonstrating the occurrence of viruses in both declining and non-declining trees and, further, identify no association between any virus and decline. ALV1 was initially hypothesized to be implicated in the rapid decline phenomenon [8] but was not detected in this study. Additionally, no association was found between decline outcome and the number of unique viruses and viroids detected in the tree. Together, our findings suggest that individual apple viruses and viroids, or coinfections thereof, are not primarily responsible for the rapid decline of young apple trees on dwarfing and semi-dwarfing rootstocks in high-density orchards. It is important to note, however, that this study focused on evaluating the role of a known set of viruses in the decline phenomenon but not of other pathogens.

The incidence of tree decline varied dramatically from year to year in both orchards evaluated in this study. Over the course of five years in the experimental orchard, tree decline was observed only during the 2022 growing season (year 4), despite the implementation of identical orchard management practices each year (apart from the manual removal of fruitlets in the first two years to promote tree establishment). We therefore hypothesized that variations in weather conditions from year to year at the experimental orchard site would correlate with the year-to-year differences in tree decline incidence. Our analyses revealed a significant rainfall deficit in mid- to late summer and early autumn 2022 and a striking negative water balance in August, September, and October 2022 despite the supplemental water provided via drip irrigation. As our weather analyses are purely observational, we cannot conclude with certainty that this water deficit was responsible for tree decline or collapse in the experimental orchard. However, Xu et al. (2023) recently documented that tree mortality of rapidly declining trees at multiple sites in British Columbia was associated with severe disruption in xylem water transport and hydraulic failure, as evidenced by reduced xylem function, reduced stomatal conductance and stem water potential [11]. It is possible that a substantial water deficit, as observed at the experimental orchard site, may have contributed to mortality in trees already experiencing water transport disruption. Conversely, at the commercial orchard site, the greatest decline incidence was observed during the growing season (2021), with the greatest cumulative rainfall during the study. Although trees at the commercial orchard site were not irrigated, the estimated orchard water balance never fell below zero over the course of the entire study. These findings suggest that either orchard water balance is not primarily responsible for the decline phenomenon or the etiologies of the decline phenomena observed in the experimental and commercial orchards are distinct.

Weather data analyses additionally revealed that the experimental orchard site experienced more extreme temperature fluctuations and a lower minimum temperature in January 2022 compared with the other years of the study. It is possible that the affected trees may have been weakened due to cold temperatures and extreme temperature fluctuations. It should be noted that in the case of the experimental orchard, weather data were obtained from an on-farm weather station located less than three kilometers from the orchard site. However, in the case of the commercial orchard site, weather data were obtained from a weather station located approximately ten kilometers from the orchard site; therefore, microclimatic variations in weather conditions at the commercial orchard site may not be well represented in the data evaluated.

As previously reported for the Wayne County commercial apple orchard by Serrano et al. (2023), the root systems of declining trees significantly differed from the root systems of their non-declining counterparts [10]. Declining trees had significantly smaller scion trunk diameter, root system width, number of primary roots, total root length, root system surface area, and root system dry weight. Other root system traits did not differ significantly between the declining and non-declining trees evaluated, including rootstock trunk diameter, the ratio of scion to rootstock trunk diameter, root system depth, length of the rootstock shank below the soil level, projected root system area, total number of root tips, maximum root diameter, and root system volume [10]. Interestingly, no significant differences between the root systems of declining vs. non-declining trees in a second orchard in eastern New York were previously found [10]. A decrease in average root system depth was noted in declining trees in our experimental orchard, although this difference was not found to be statistically significant when a *p* value threshold of 0.05 was applied. These findings suggest that either root system architecture is not primarily responsible for the decline phenomenon or the etiologies of the decline phenomena observed in the experimental and commercial orchards are distinct.

A growing body of evidence is pointing toward vascular transport disruption as a cause of the gradual decline of apple trees, suggesting vascular failure as the immediate cause of tree collapse [10,11]. However, it remains unclear what factors may be initiating this vascular transport disruption. More work is needed to address these issues in experimental orchards and elucidate the specific triggers of the decline phenomenon. Ideally, uninfected trees should be used as negative controls in such studies. If the involvement of tree water balance, water stress response, crop load, or root system architecture in decline is confirmed, horticultural mitigation, such as minimizing water stress, would appear to be a compelling option for preventing apple decline. We hope that future studies will elucidate the specific triggers of the decline phenomenon to inform more precise management strategies.

## 4. Materials and Methods

### 4.1. Plant Material and Virus Inoculation

Standard nonfeathered nursery trees representing six scion-rootstock combinations (‘Baigent’ Gala, ‘Honeycrisp’, and ‘Royal Red Honeycrisp’™ on ‘Malling 26’ or ‘Geneva 935’ rootstocks) were custom-budded in 2018 and chip graft inoculated with either apple chlorotic leaf spot virus (ACLSV), apple stem pitting virus (ASPV), both viruses, or neither virus. Briefly, a thin segment of bark was excised from the stem of the test plant, exposing the cambium, and then replaced with a segment of bark and cambium tissue of equivalent dimensions from an infected source tree selected based on previous virus diagnosis via double-antibody sandwich enzyme-linked immunosorbent assay (DAS-ELISA) with specific antibodies (BIOREBA, Reinach, Switzerland). Chip grafts were moistened with water, wrapped with grafting tape, and allowed to heal. The initial experimental design consisted of a full factorial design of three scion cultivars, two rootstock genotypes, and four virus treatments comprising 24 combinations. Ten replicates of each of the 24 combinations were produced for a total of 240 trees.

### 4.2. Experimental Orchard Establishment and Management

An experimental orchard comprising 240 trees was established in June 2019 at Cornell AgriTech in Ontario County, NY, USA, on a Lima series well-drained loam soil [20]. Following bareroot storage at 4 °C, trees were established at the orchard site in a tall spindle production system at 0.9 m × 3.4 m high-density spacing (equivalent to approximately 3260 trees per hectare) according to a randomized complete block design. Rainfall was supplemented with approximately 0.6 cm of water per week by drip irrigation throughout each growing season (May–September), and insecticide, fungicide, and antibiotic applications were made as needed throughout the course of the study for pest and disease management. Any observed fire blight cankers (shoot infections by *E. amylovora*) were pruned out at least 30 cm below the symptomatic tissue using pruning shears disinfected with 2.5% sodium hypochlorite between each pruning cut, and trees were removed if fire blight reached the central leader.

Trees were supported by a three-wire trellis (2.7 m), and the tall spindle production system was maintained by annual winter pruning to remove branches competing with the central leader and lateral branches with diameters greater than 2 cm. Leaders were not headed for the duration of the study. Fruitlets produced by trees during the first two years following orchard establishment (2019 and 2020) were manually removed to encourage successful tree establishment. For the remainder of the study (2021 and 2022), trees were allowed to crop, and fruitlets were manually thinned each spring to approximately two fruits per cluster according to standard practices for conventional tall spindle apple production in the region.

### 4.3. Commercial Orchard Site Selection

A high-density orchard block of ‘Honeycrisp’ apple trees on ‘Malling 9 Nic29’ rootstocks established in 2018 was selected for this study after the orchardist first noticed suddenly declining trees in 2020 (year 3). This commercial block was located in Wayne County, NY, USA, in the Lake Ontario apple production region. Trees were established at the orchard site in a tall spindle production system at 1.25 m × 3 m high-density spacing with trellis support. Conventional horticultural and pest management practices for apple orchards in the region were applied throughout the duration of this work. The orchard block was not irrigated.

### 4.4. Assessment of Tree Decline Severity

Trees in the experimental orchard at Cornell AgriTech were visually evaluated for decline during the 2020–2023 growing seasons using a scale from 0 to 4, with a rating of 0 indicating no decline symptoms and a rating of 4 indicating tree mortality. Ratings of 1 to 3 indicate increasing decline severity, with 1 corresponding to chlorosis throughout <50% of the canopy, 2 to chlorosis throughout ≥50% of the canopy, and 3 to chlorosis throughout ≥50% of the canopy, leaf flagging, and poor tree vigor. Prior to tree excavation for analysis of root system architecture in late autumn 2022, trees were grouped into two categories: a non-declining category comprised of trees with severity ratings of 0 and 1 and a declining category comprised of trees with severity ratings of 2, 3, and 4 as previously described [10].

Trees in the commercial orchard block in Wayne County were visually evaluated for decline during the 2021–2023 growing seasons using the same 0 to 4 rating. 

### 4.5. Tissue Sampling and Virus Detection from Declining and Non-Declining Trees

Leaf and root tissue samples of declining and non-declining trees in the experimental orchard at Cornell AgriTech were screened for 19 viruses and viroids of pome fruit trees via multiplex PCR-based amplicon sequencing as previously described [21,22]. Briefly, a leaf sample consisting of 5–6 mature leaves from throughout the scaffold and a composite root tissue sample consisting of fine roots excavated from the top 15 cm of soil surrounding the trunk were collected from each tree in the summer of 2022. Then, approximately 100 mg of each tissue sample was homogenized in a guanidine thiocyanate and β-mercaptoethanol lysis buffer with two steel beads (4.5 mm diameter) using a Retsch MM400 mixer mill (Retsch, Haan, Germany) at 30 Hz for 70 s (leaf tissue) or 180 s (root tissue). Next, total RNA was isolated using the GenCatch Plant Total RNA Miniprep Kit (Epoch Life Science, Missouri City, TX, USA) following the manufacturers’ guidelines, quantified using a Qubit 2.0 Fluorometer (ThermoFisher Scientific, Waltham, MA, USA), and stored at −80 °C prior to virus screening. Each sample was tested for the presence of 14 viruses and five viroids of pome fruit trees via multiplex PCR-based amplicon sequencing as described by Costa et al. [21]. Namely, each sample was tested for the presence of the alphapartitivirus Pyrus pyrifolia partitivirus 2 (PpPV2), the capillovirus ASGV, the coguviruses citrus concave gum-associated virus (CCGaV) and citrus virus A (CiVA), the foveaviruses apple green crinkle-associated virus (AGCaV) and ASPV, the ilarvirus apple mosaic virus (ApMV), the nepoviruses tobacco ringspot virus (TRSV) and tomato ringspot virus (ToRSV), the rubodviruses apple rubbery wood virus 1 (ARWV1) and apple rubbery wood virus 2 (ARWV2), the tepovirus Prunus virus T (PrVT), the tombusvirus apple luteovirus 1 (ALV1), the trichovirus ACLSV, the apscaviroids apple dimple fruit viroid (ADFVd), apple fruit crinkle viroid (AFCVd), apple scar skin viroid (ASSVd), and pear blister canker viroid (PBCVd), and the pelamoviroid apple hammerhead viroid (AHVd).

### 4.6. Foliar Nutrient Analysis of Declining and Non-Declining Trees

Foliar nutrient analysis was conducted for declining and non-declining trees in the Wayne County commercial orchard in August 2021. Briefly, approximately six leaves were collected from current season terminal shoots from throughout the canopies of each of ten trees corresponding to each decline severity category 0–3 described above. Leaf tissue samples were not collected from trees representing decline severity category 4 (tree mortality). Bulked leaf samples comprising 60–100 leaves were then prepared for each of the four decline severity rating categories and submitted to DairyOne Forage Laboratory (Ithaca, NY, USA) for nutrient analysis via microwave digestion and inductively coupled plasma optical emission spectroscopy. Nutrient concentration thresholds were assigned according to Pennsylvania State University nutrient guidelines for cultivated apple [19].

### 4.7. Analysis of Weather Data and Orchard Water Balance

Analysis of weather patterns at the experimental orchard site at Cornell AgriTech was conducted using data obtained from an on-farm weather station located less than three kilometers from the orchard site via Cornell University’s Network for Environment and Weather Applications (NEWA). Analysis of weather patterns at the commercial orchard site in Wayne County was conducted using data obtained from a weather station located approximately ten kilometers from the orchard site via NEWA.

Water balance analyses of the experimental orchard and commercial orchard sites were conducted using the NEWA Apple Irrigation Model [23] using the appropriate green tip dates, orchard ages, and tree spacing parameters for each orchard.

### 4.8. Root System Sampling, Processing, and Analysis

Trees in the experimental orchard at Cornell AgriTech were assigned to one of two decline categories based on their decline severity ratings in October 2022 as described above declining (decline severity ratings 2, 3, and 4) and non-declining (decline severity ratings 0 and 1). Representative trees from each of the two categories were excavated in October 2022 to facilitate root system architecture analysis. The aboveground portion of each tree was cut approximately 30 cm above the graft union and removed. An excavator was then used to remove the soil from around the trunk of each tree in a radius equal to half the tree spacing (approximately 45 cm) and to a depth of approximately 35 cm. After removing loose soil, the lower portion of each tree, including the portion of the root system within the excavation zone, was removed and stored at 4 °C prior to processing and evaluation of root traits.

Each of the excavated root systems was cleaned with tap water and suspended from a horizontal metal rail by threading a screw into the trunk to facilitate imaging as previously described [10]. Four images of each root system were captured using a Nikon D850 camera (Nikon, Minato, Tokyo, Japan) by rotating the root system in 90° increments. Images were analyzed using ImageJ version 1.54d [24] as previously described [10]. Root system architecture parameters evaluated included scion trunk diameter at the graft union, rootstock trunk diameter at the graft union, ratio of rootstock trunk diameter to scion trunk diameter, rootstock shank length below the soil level, root system depth, root system width, and projected area of the root system.

Similarly, trees representative of both the declining and non-declining categories were excavated from the commercial orchard block in Wayne County in November 2021. Root system sampling, processing, and analysis were conducted as previously described [10].

### 4.9. Statistical Analysis

Statistical analyses were performed using R statistical software (v4.3.1; R Core Team 2023). Chi-squared tests of independence with Bonferroni adjustments were conducted to identify significant associations between decline outcome and virus presence. Type III analysis of variance tests were conducted to evaluate whether root system architecture traits differed by tree decline outcome using the ‘car’ statistical software package in R (v3.1-3) [25]. Tukey’s Honest Significant Difference tests were conducted to assess the significance of differences between group means using the ‘agricolae’ statistical software package in R (v1.3-7) [26].

## Figures and Tables

**Table 1:** Viruses and viroids detected via multiplex PCR-based amplicon sequencing of 175 three-year-old ‘Baigent’ Gala, ‘Honeycrisp’, and ‘Royal Red Honeycrisp’™ apple trees on ‘Malling 26’ and ‘Geneva 935’ rootstocks in a high-density experimental orchard at Cornell AgriTech in Ontario County, NY, USA. Numbers of infected trees per virus or viroid are indicated.

	Rootstock/Scion						
Virus	G.935/Gala	G.935/HC	G.935/RRHC	M.26/Gala	M.26/HC	M.26/RRHC	Total
ACLSV	21	22	30	21	34	8	136
AGCaV	13	7	14	11	7	4	56
ALV1	0	0	0	0	0	0	0
ApMV	0	0	0	0	0	0	0
ARWV1	0	0	0	0	0	0	0
ARWV2	2	2	15	1	5	0	25
ASGV	31	34	30	33	39	8	175
ASPV	21	19	30	21	26	8	125
CCGaV	17	19	29	12	22	7	106
CiVA	0	0	0	0	0	0	0
PpPV2	0	0	0	0	0	0	0
PrVT	0	0	0	0	0	0	0
ToRSV	0	0	2	1	0	0	3
TRSV	0	0	0	0	1	0	1
Viroid	G.935/Gala	G.935/HC	G.935/RRHC	M.26/Gala	M.26/HC	M.26/RRHC	Total
ADFVd	0	0	0	0	0	0	0
AFCVd	0	0	0	0	0	0	0
AHVd	29	28	30	8	6	3	104
ASSVd	0	0	0	0	0	0	0
PBCVd	0	0	0	0	0	0	0

Abbreviations: ACLSV, apple chlorotic leaf spot virus; ADFVd, apple dimple fruit viroid; AFCVd, apple fruit crinkle viroid; AGCaV, apple green crinkle-associated virus; AHVd, apple hammerhead viroid; ALV1, apple luteovirus 1; ApMV, apple mosaic virus; ARWV1, apple rubbery wood virus 1; ARWV2, apple rubbery wood virus 2; ASGV, apple stem grooving virus; ASPV, apple stem pitting virus; ASSVd, apple scar skin viroid; CCGaV, citrus concave gum-associated virus; CiVA, citrus virus A; Gala, ‘Baigent’ Gala; G.935, ‘Geneva 935’; HC, ‘Honeycrisp’; M.26, ‘Malling 26’; PBCVd, pear blister canker viroid; PpPV2, Pyrus pyrifolia partitivirus 2; PrVT, Prunus virus T; RRHC, ‘Royal Red Honeycrisp’™; ToRSV, tomato ringspot virus; TRSV, tobacco ringspot virus.

**Table 2:** Root system architecture traits of declining (decline severity ratings 2, 3, and 4) and non-declining (decline severity ratings 0 and 1) three-year-old ‘Baigent’ Gala, ‘Honeycrisp’, and ‘Royal Red Honeycrisp’™ apple trees on ‘Malling 26’ and ‘Geneva 935’ rootstocks in a high-density experimental orchard at Cornell AgriTech in Ontario County, NY, USA.

	Declining		Non-Declining				
Number of Trees	25		14				
Parameter	Mean ^a^	SD	Mean ^a^	SD	Decline *p* Value ^b^	Decline × Cultivar *p* Value ^c^	Decline × Rootstock *p* Value ^c^
ØScion (cm)	3.58 a	0.71	3.57 a	0.58	0.940	0.136	0.230
ØRootstock (cm)	4.72 a	0.83	4.61 a	0.82	0.667	0.179	0.472
ØR/ØS (cm)	1.33 a	0.15	1.30 a	0.18	0.552	0.997	0.140
RootstockUG (cm)	25.81 a	10.06	28.91 a	5.83	0.228	0.835	0.351
RSDepth (cm)	53.55 a	14.85	63.67 a	15.82	0.058 ^†^	0.431	0.953
RSWidth (cm)	73.44 a	18.77	68.91 a	15.62	0.424	0.214	0.800
RSArea (cm^2^)	4281.49 a	1487.92	4314.82 a	1715.49	0.952	0.716	0.867

Root system parameters evaluated were scion trunk diameter at the graft union (ØScion), rootstock trunk diameter at the graft union (ØRootstock), ratio of rootstock trunk diameter to scion trunk diameter (ØR/ØS), rootstock shank length below the soil level (RootstockUG), root system depth (RSDepth), root system width (RSWidth), and projected area of the root system (RSArea). SD indicates standard deviations. ^a^ Means followed by the same letter in both columns are not significantly different according to Tukey’s Honest Significant Difference test with a *p* value threshold of 0.05. ^b^ *p* values indicate statistical significance of the interactions between root system trait and decline outcome according to one-way ANOVA with a *p* value threshold of 0.05. ^c^ *p* values indicate the statistical significance of the interactions between root system trait, decline status, and cultivar or rootstock according to two-way ANOVA with a *p* value threshold of 0.05. ^†^ *p* < 0.10.

## Data Availability

The original contributions presented in the study are included in the article and the Supplementary Materials; further inquiries can be directed to the corresponding author.

## Supplementary Materials

The following supporting information can be downloaded at https://www.mdpi.com/article/10.3390/plants13202866/s1, Table S1: Virus and viroid status of 175 three-year-old ‘Baigent’ Gala, ‘Honeycrisp’, and ‘Royal Red Honeycrisp’™ apple trees on ‘Malling 26’ and ‘Geneva 935’ rootstocks in a high-density experimental orchard at Cornell AgriTech in Ontario County, NY, USA; Table S2: Root system architecture traits by cultivar (‘Baigent’ Gala or ‘Honeycrisp’) of declining (decline severity ratings 2, 3, and 4) and non-declining (decline severity ratings 0 and 1) three-year-old apple trees in a high-density experimental orchard at Cornell AgriTech in Ontario County, NY, USA; Table S3: Root system architecture traits by rootstock (‘Geneva 935’ or ‘Malling 26’) of declining (decline severity ratings 2, 3, and 4) and non-declining (decline severity ratings 0 and 1) three-year-old apple trees in a high-density experimental orchard at Cornell AgriTech in Ontario County, NY, USA.
