# Supplementary material for: Investigating the Role of Viruses in the Rapid Decline of Young Apple Trees in High-Density Orchards in New York
Source: Plants (Basel). 2024 Oct 14;13(20):2866. doi: 10.3390/plants13202866 (PMC11511006; doi:10.3390/plants13202866)
Supplement: Supplementary file 1 [file plants-13-02866-s001.zip › Table S1.pdf]

Table S1. Virus and viroid status of 175 three-year-old 'Baigent' Gala, 'Honeycrisp', and 'Royal Red Honeycrisp'™ apple trees on 'Malling 26' and 'Geneva 935' rootstocks in a high-density experimental orchard at Cornell AgriTech in Ontario County, NY, USA.

Virus and viroid status[a]	Number of trees	Non-declining (0 – 1)[b]	Declining (2 – 3)[b]	Collapsed (4)[b]
ASGV	18	10/18 (56%)	2/18 (11%)	6/18 (33%)
ASGV + AHVd	18	18/18 (100%)	0/18 (0%)	0/18 (0%)
ASGV + ACLSV	3	0/3 (0%)	3/3 (100%)	0/3 (0%)
ASGV + CCGaV	1	1/1 (100%)	0/1 (0%)	0/1 (0%)
ASGV + ToRSV	1	0/1 (0%)	0/1 (0%)	1/1 (100%)
ASGV + AHVd + ACLSV	1	1/1 (100%)	0/1 (0%)	0/1 (0%)
ASGV + AHVd + CCGaV	1	1/1 (100%)	0/1 (0%)	0/1 (0%)
ASGV + ACLSV + ASPV	5	4/5 (80%)	1/5 (20%)	0/5 (0%)
ASGV + ACLSV + CCGaV	3	2/3 (67%)	0/3 (0%)	1/3 (33%)
ASGV + AHVd + ACLSV + ASPV	3	3/3 (100%)	0/3 (0%)	0/3 (0%)
ASGV + AHVd + ACLSV + CCGaV	3	2/3 (67%)	1/3 (33%)	0/3 (0%)
ASGV + ACLSV + ASPV + CCGaV	22	12/22 (55%)	3/22 (14%)	7/22 (32%)
ASGV + ACLSV + ASPV + ACGaV	8	3/8 (38%)	0/8 (0%)	5/8 (63%)
ASGV + ACLSV + ASPV + ARWV2	1	1/1 (100%)	0/1 (0%)	0/1 (0%)
ASGV + ACLSV + ASPV + TRSV	1	0/1 (0%)	0/1 (0%)	1/1 (100%)
ASGV + ACLSV + CCGaV + ARWV2	1	1/1 (100%)	0/1 (0%)	0/1 (0%)
ASGV + ACLSV + ASPV + AHVd + CCGaV	24	16/24 (67%)	5/24 (21%)	2/24 (8.3%)
ASGV + ACLSV + ASPV + AHVd + AGCaV	10	9/10 (90%)	0/10 (0%)	1/10 (10%)
ASGV + ACLSV + ASPV + CCGaV + ARWV2	2	0/2 (0%)	0/2 (0%)	2/2 (100%)
ASGV + ACLSV + ASPV + CCGaV + AGCaV	5	1/5 (20%)	1/5 (20%)	3/5 (60%)
AGSV + AHVd + ACLSV + ASPV + CCGaV + ARWV2	11	6/11 (55%)	4/11 (36%)	1/11 (9.1%)
AGSV + AHVd + ACLSV + ASPV + CCGaV + AGCaV	21	21/21 (100%)	0/21 (0%)	0/21 (0%)
ASGV + AHVd + ASGV + ASPV + CCGaV + ARWV2 + AGCaV	10	10/10 (100%)	0/10 (0%)	0/10 (0%)
AGSV + AHVd + ACLSV + ASPV + CCGaV + AGCaV + TRSV	2	2/2 (100%)	0/2 (0%)	0/2 (0%)

[a]Virus and viroid status was determined via multiplex PCR-based amplicon sequencing of tissue samples collected in June 2022.

[b]Non-declining trees received decline severity ratings of either 0 or 1, declining trees received decline severity ratings of either 2 or 3, and collapsed trees received decline severity ratings of 4 in October 2022.

Abbreviations: ACLSV, apple chlorotic leaf spot virus; AGCaV, apple green crinkle-associated virus; AHVd, apple hammerhead viroid; ARWV2, apple rubbery wood virus 2; ASGV, apple stem grooving virus; ASPV, apple stem pitting virus; CCGaV, citrus concave gum-associated virus; ToRSV, tomato ringspot virus; TRSV, tobacco ringspot virus.
