# Supplementary material for: Investigating the Role of Viruses in the Rapid Decline of Young Apple Trees in High-Density Orchards in New York
Source: Plants (Basel). 2024 Oct 14;13(20):2866. doi: 10.3390/plants13202866 (PMC11511006; doi:10.3390/plants13202866)
Supplement: Supplementary file 1 [file plants-13-02866-s001.zip › Table S2.pdf]

Table S2. Root system architecture traits by cultivar ('Baigent' Gala or 'Honeycrisp') of declining (decline severity ratings 2, 3, and 4) and non-declining (decline severity ratings 0 and 1) three-year-old apple trees in a high-density experimental orchard at Cornell AgriTech in Ontario County, NY, USA.

	**Brookfield® 'Gala'**				**'Honeycrisp'**				
	**Declining**		**Non-declining**		**Declining**		**Non-declining**		
Number of trees	7		12		6		9		
**Parameter**	Mean[a]	SD	Mean[a]	SD	Mean[a]	SD	Mean[a]	SD	*p* value[b]
ØScion (cm)	3.99a	0.64	3.89ab	0.53	3.05b	0.48	3.39ab	0.39	0.001**
ØRootstock (cm)	5.14a	0.86	4.92a	0.77	4.13a	0.36	4.45a	0.67	0.025*
ØR/ØS (cm)	1.30a	0.19	1.27a	0.14	1.37a	0.12	1.32a	0.24	0.568
RootstockUG (cm)	24.79a	9.69	29.22a	6.03	26.67a	12.13	29.57a	6.29	0.909
RSDepth (cm)	46.39a	13.16	61.88a	17.57	58.32a	13.18	63.26a	14.48	0.174
RSWidth (cm)	81.85a	18.71	69.38a	17.23	60.83a	12.24	67.07a	14.93	0.213
RSArea (cm^2)	4326.08a	1307.16	4075.59a	1747.02	3835.61a	1532.15	4152.76a	1486.17	0.133

Root system parameters evaluated were scion trunk diameter at the graft union (ØScion), rootstock trunk diameter at the graft union (ØRootstock), ratio of rootstock trunk diameter to scion trunk diameter (ØR/ØS), rootstock shank length below the soil level (RootstockUG), root system depth (RSDepth), root system width (RSWidth), and projected area of the root system (RSArea). SD indicates standard deviations. Root system traits of 'Royal Red Honeycrisp'™ trees were not compared between declining (decline severity ratings 2, 3, and 4) and non-declining trees (decline severity ratings 0 and 1) due to insufficient sample sizes.

[a]Means followed by the same letter are not significantly different according to Tukey's Honest Significant Difference test with a *p* value threshold of 0.05.

[b]*p* values indicate statistical significance of the interactions between root system trait and cultivar according to one-way ANOVA with a *p* value threshold of 0.05.
* $p < 0.05$; ** $p < 0.01$.
