# Supplementary material for: Investigating the Role of Viruses in the Rapid Decline of Young Apple Trees in High-Density Orchards in New York
Source: Plants (Basel). 2024 Oct 14;13(20):2866. doi: 10.3390/plants13202866 (PMC11511006; doi:10.3390/plants13202866)
Supplement: Supplementary file 1 [file plants-13-02866-s001.zip › Table S3.pdf]

Table S3. Root system architecture traits by rootstock ('Geneva 935' or 'Malling 26') of declining (decline severity ratings 2, 3, and 4) and non-declining (decline severity ratings 0 and 1) three-year-old apple trees in a high-density experimental orchard at Cornell AgriTech in Ontario County, NY, USA.

	**'Geneva 935'**				**'Malling 26'**				
	**Declining**		**Non-declining**		**Declining**		**Non-declining**		
Number of trees	7		12		6		9		
**Parameter**	Mean[a]	SD	Mean[a]	SD	Mean[a]	SD	Mean[a]	SD	p value[b]
ØScion (cm)	4.00a	0.68	3.58a	0.54	3.41a	0.68	3.56a	0.62	0.322
ØRootstock (cm)	4.77a	0.53	4.95a	0.77	4.71a	0.94	4.44a	0.82	0.227
ØR/ØS (cm)	1.21a	0.19	1.39a	0.22	1.38a	0.11	1.26a	0.15	0.617
RootstockUG (cm)	22.86a	11.02	29.72a	4.99	26.99a	10.01	28.53a	6.30	0.847
RSDepth (cm)	55.86a	19.02	66.33a	17.63	52.63a	13.94	62.41a	15.30	0.475
RSWidth (cm)	72.92a	19.42	66.23a	16.43	73.64a	19.57	70.17a	15.57	0.612
RSArea (cm^2)	4113.38a	1861.52	4014.01a	1710.40	4348.73a	1423.09	4456.38a	1751.55	0.518

Root system parameters evaluated were scion trunk diameter at the graft union (ØScion), rootstock trunk diameter at the graft union (ØRootstock), ratio of rootstock trunk diameter to scion trunk diameter (ØR/ØS), rootstock shank length below the soil level (RootstockUG), root system depth (RSDepth), root system width (RSWidth), and projected area of the root system (RSArea). SD indicates standard deviations.

[a]Means followed by the same letter are not significantly different according to Tukey's Honest Significant Difference test with a p value threshold of 0.05.
[b]p values indicate statistical significance of the interactions between root system trait and rootstock according to one-way ANOVA with a p value threshold of 0.05.
